# Complete Genome Sequence of a Potential Novel *Bacillus* sp. Strain, FJAT-18017, Isolated from a Potato Field

**DOI:** 10.1128/genomeA.01424-16

**Published:** 2017-01-19

**Authors:** Guo-Hong Liu, Bo Liu, Jie-Ping Wang, Jian-Mei Che, Qian-Qian Chen

**Affiliations:** Agricultural Bio-Resource Institute, Fujian Academy of Agricultural Sciences, Fuzhou, Fujian, China

## Abstract

*Bacillus* sp. strain FJAT-18017 was isolated from a potato field in Xinjiang, China. This paper is the first report, to our knowledge, to demonstrate the fully sequenced and completely annotated genome of *Bacillus* sp. FJAT-18017. The genome size is 5,265,521 bp. The average G+C content was 42.42%.

## GENOME ANNOUNCEMENT

During a survey of *Bacillus* diversity in Xinjiang, China, we found one potential species belonging to the genus *Bacillus*, designated strain FJAT-18017, which was isolated from potato rhizosphere soil in Xinjiang.

We determined the complete genome sequence of *Bacillus* sp. FJAT-18017 using a whole-genome shotgun strategy using Sanger sequencing (Illumina HiSeq 2500 system). DNA libraries with insert sizes of 500 bp were constructed and sequenced using the 2 × 150-bp paired-end sequencing strategy. After filtering of the 1.22 Gb of raw data, 1.12 Gb of clean data were obtained, providing approximately 200-fold coverage. Through the data assembly, one scaffold with total length of 5,265,521 bp was obtained. A total of 93.32% clean reads could be aligned back to the genome, which covered 99.18% of the sequence. The reads were assembled via the SOAP*denovo* software version 1.05 (1), using a key parameter K setting at 77. The annotation of the genome was performed using the NCBI Prokaryotic Genome Annotation Pipeline (PGAP) (http://www.ncbi.nlm.nih.gov/genomes/static/Pipeline.html) utilizing the GeneMark, Glimmer, and tRNAscan-SE tools (2).

The genome sequence of *Bacillus* sp. FJAT-18017 consists of a circular chromosome of 5,265,521 bp with no plasmid. There are 4,950 genes predicted, including 4,637 coding sequences (CDSs), 197 pseudogenes, 30 rRNA operons, 85 tRNAs, and 36 frameshifted genes. *Bacillus* sp. FJAT-18017 is related to *Bacillus subterraneus* MITOT1 in the phylogenetic tree of the family *Bacillaceae*. Then, we compared the genome of FJAT-18017 with that (5,265,521 bp) of *B. subterraneus* MITOT1 (accession no. JXIQ00000000) (3). There were 3,346 and 2,418 genes assigned to the COG and KEGG databases, respectively. The average G+C content was 42.42%. This paper is the first report demonstrating the fully sequenced and completely annotated genome of *Bacillus* sp. FJAT-18017. The genome information of this species will be useful for further studies of its physiology, taxonomy, and ecology.

### Accession number(s).

The sequence data for the genome have been deposited in DDBJ/GenBank/EMBL under the GenBank accession no. CP012602. The version described in this paper is the first version.

## References

[1] Li R, Zhu H, Ruan J, Qian W, Fang X, Shi Z, Li Y, Li S, Shan G, Kristiansen K, Li S, Yang H, Wang J, Wang J 2010 *De novo* assembly of human genomes with massively parallel short read sequencing. Genome Res 20:265–272. doi:10.1101/gr.097261.109. 20019144 PMC2813482

[2] Aziz RK, Bartels D, Best AA, DeJongh M, Disz T, Edwards RA, Formsma K, Gerdes S, Glass EM, Kubal M, Meyer F, Olsen GJ, Olson R, Osterman AL, Overbeek RA, McNeil LK, Paarmann D, Paczian T, Parrello B, Pusch GD, Reich C, Stevens R, Vassieva O, Vonstein V, Wilke A, Zagnitko O 2008 The RAST server: rapid annotations using subsystems technology. BMC Genomics 9:75. doi:10.1186/1471-2164-9-75. 18261238 PMC2265698

[3] Peet KC, Thompson JR 2015 Draft genome sequences of supercritical CO_2_-tolerant bacteria *Bacillus subterraneus* MITOT1 and *Bacillus cereus* MIT0214. Genome Announc 3(2):e00140-15. doi:10.1128/genomeA.00140-15. 25858826 PMC4392138

